# MicroRNA 322 Aggravates Dexamethasone-Induced Muscle Atrophy by Targeting *IGF1R* and *INSR*

**DOI:** 10.3390/ijms21031111

**Published:** 2020-02-07

**Authors:** Hongwei Geng, Qinglong Song, Yunyun Cheng, Haoyang Li, Rui Yang, Songcai Liu, Linlin Hao

**Affiliations:** 1 College of Animal Science, Jilin University, Changchun 130062, China; genghw_jlu@163.com (H.G.); chengyy@scau.edu.cn (Y.C.); hyli17@mails.jlu.edu.cn (H.L.); songcai@jlu.edu.cn (S.L.); 2 State Key Laboratory of Animal Nutrition, China Agricultural University, Beijing 100193, China; songql@nferc.org; 3 Beijing Key Laboratory of Bio-Feed Additives, Beijing 100193, China; 4 Five-Star Animal Health Pharmaceutical Factory of Jilin Province, Changchun 130062, China

**Keywords:** miR-322, dexamethasone, IGF1R, INSR, atrophy, skeletal muscle

## Abstract

Dexamethasone (Dex) has been widely used as a potent anti-inflammatory, antishock, and immunosuppressive agent. However, high dose or long-term use of Dex is accompanied by side effects including skeletal muscle atrophy, whose underlying mechanisms remain incompletely understood. A number of microRNAs (miRNAs) have been shown to play key roles in skeletal muscle atrophy. Previous studies showed significantly increased miR-322 expression in Dex-treated C2C12 myotubes. In our study, the glucocorticoid receptor (*GR*) was required for Dex to increase miR-322 expression in C2C12 myotubes. miR-322 mimic or miR-322 inhibitor was used for regulating the expression of miR-322. Insulin-like growth factor 1 receptor (*IGF1R*) and insulin receptor (*INSR*) were identified as target genes of miR-322 using luciferase reporter assays and played key roles in Dex-induced muscle atrophy. miR-322 overexpression promoted atrophy in Dex-treated C2C12 myotubes and the gastrocnemius muscles of mice. Conversely, miR-322 inhibition showed the opposite effects. These data suggested that miR-322 contributes to Dex-induced muscle atrophy via targeting of *IGF1R* and *INSR*. Furthermore, miR-322 might be a potential target to counter Dex-induced muscle atrophy. miR-322 inhibition might also represent a therapeutic approach for Dex-induced muscle atrophy.

## 1. Introduction

Dexamethasone (Dex) is an effective synthetic glucocorticoid. And it is commonly used as a therapeutic agent due to its potent anti-inflammatory, antishock, and immunosuppressive functions [1,2]. However, Dex also causes passive effects, including skeletal muscle atrophy [3,4]. Muscle atrophy decreases the quality of life of patients and increases their risk of mortality. However, the treatment of muscle atrophy remains an unresolved challenge [5]. Therefore, developing novel therapies to combat Dex-induced muscle atrophy is urgently needed [6]. Previous studies have shown that Dex exhibits direct effects on decreasing protein synthesis and increasing protein catabolism, thereby leading to muscle atrophy [7,8]. Dex-treated C2C12 myotubes and skeletal muscle have been widely used as models of muscle atrophy to explore the potential mechanisms of Dex [9,10]. The insulin/insulin-like growth factor 1 (*IGF1*) signaling pathway plays a crucial role in the dysregulation of muscle protein turnover [11,12]. Downregulation of insulin/*IGF1* signaling inhibits thymoma viral proto-oncogene 1 (*Akt*) phosphorylation, leading to activation of forkhead box O1 (*FOXO1*) proteins, which increases the transcription of two ubiquitin E3 ligases, namely *MuRF-1* and *Atrogin-1*, and other atrophy-related genes [13,14,15].

MicroRNAs (miRNAs) are small endogenous noncoding RNA molecules that negatively regulate gene expression by targeting sequences in the 3′-untranslated region (3′-UTR) of mRNAs [16]. In previous studies, miRNAs have been reported to play fundamental roles in diverse biological and pathological processes of muscles [17,18,19]. For example, miR-29b overexpression sufficiently promotes muscle atrophy, whereas miR-29b inhibition attenuates muscle atrophy by targeting *IGF1* and phosphoinositide 3-kinase (p85a) [10]. Moreover, a decrease in miR-23a results in increased translation of *TRIM63*/*MuRF-1* and *FBXO32*/*Atrogin-1*, whereas miR-23a overexpression protects muscles from glucocorticoid-induced skeletal muscle atrophy [20,21]. miRNA microarray was also used to investigate the differential expression of miRNAs in C2C12 myotubes with and without Dex treatment. The results showed significantly increased miR-322 expression in Dex-treated C2C12 myotubes [22]. miR-322 is mammal-specific member of the extended miR-15/107 microRNA family, and it regulates fundamental cellular processes, including cell cycle, epithelial-to-mesenchymal transition, hypoxia, and other stress responses [23]. A previous study suggested that miR-322 targeted *IGF1R* and *INSR* to regulate the signaling pathway involved in mitochondrial function and fatty acid oxidation in the heart of mice [24]. In addition, miR-322 was reported to control skeletal muscle differentiation [25].

In this study, we determined whether miR-322 contributes to Dex-induced muscle atrophy. Firstly, we examined the expression of miR-322. Glucocorticoid receptor (*GR*), which is also a Dex receptor, was investigated for its role in the expression of miR-322. Then, we investigated the effects of miR-322 on muscle atrophy in Dex-treated C2C12 myotubes and identified the target genes of miR-322. Finally, we investigated the effects of miR-322 on muscle atrophy in Dex-treated mice. Our data may provide theoretical support for the use of miR-322 as a novel therapeutic target for Dex-induced muscle atrophy.

## 2. Results

### 2.1. miR-322 Increased in Dex-Induced C2C12 Myotubes

**Figure 1:**
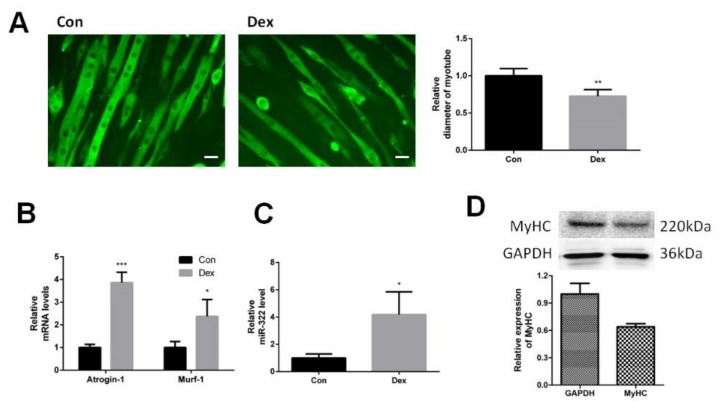
miR-322 was increased in Dex-induced C2C12 myotubes. (**A**) Immunofluorescent staining for myosin heavy chain (*MyHC*) in C2C12 myotubes showed that Dex reduced the myotube diameter of C2C12 myotubes. (**B**) Quantitative reverse transcription–PCR (qRT-PCR) analysis showed the elevated expressions of *Atrogin-1* and *MuRF-1* in C2C12 myotubes at 24 h after Dex treatment. (**C**) qRT-PCR analysis revealed the increased miR-322 expression in C2C12 myotubes at 24 h after Dex treatment. Scale bar, 10 µm. Con, control. Dex, dexamethasone. (**D**) Western blot showed decreased the expression of MyHC in C2C12 myotubes at 24 h after Dex treatment. * *p* < 0.05, ** *p* < 0.01, and *** *p* < 0.001.

C2C12 myotubes were differentiated from C2C12 myoblasts and were induced to atrophy with Dex. Immunofluorescent staining for myosin heavy chain (*MyHC*) showed the shape of C2C12 myotubes and Dex reduced the diameter of the C2C12 myotubes (Figure 1A). Dex elevated the expression of *Atrogin-1* and *MuRF-1*, marker genes of muscle atrophy, at 24 h after Dex treatment (Figure 1B). This suggested that Dex-induced atrophy in C2C12 myotubes. The expression of miRNA-322 was investigated in Dex-induced C2C12 myotubes. The results showed that Dex increased the expression of miRNA-322 (Figure 1C).

### 2.2. Dex Required GR to Increase the miR-322 Expression in C2C12 Myotubes

**Figure 2:**
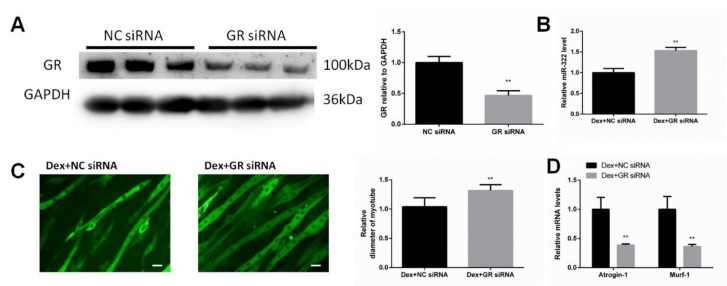
Glucocorticoid receptor (*GR*) negatively regulates miR-322 atrophy in C2C12 myotubes. (**A**) Western blot analysis showed that *GR* siRNA decreased the *GR* expression in Dex-induced C2C12 myotubes. (**B**) qRT-PCR analysis revealed the increased miR-322 expression when the C2C12 myotubes were transfected with *GR* siRNA. (**C**) Immunofluorescent staining C2C12 myotubes showed the decreased myotube diameter after transfection with *GR* siRNA. (**D**) qRT-PCR analysis revealed the increased *Atrogin-1* and *MuRF-1* expression levels when the C2C12 myotubes were transfected with *GR* siRNAs. Scale bar, 10 µm. NC, negative control. Dex, dexamethasone. ** *p* < 0.01.

*GR* is considered as the receptor of Dex. We investigated whether GR was necessary for Dex to increase the miR-322 expression in C2C12 myotubes. The siRNA for inhibiting the expression of *GR* (*GR* siRNA) was used to inhibit the *GR* expression (Figure 2A). Importantly, we observed that *GR* knockdown inhibited Dex to increase the miR-322 expression in C2C12 myotubes (Figure 2B). *GR* siRNA increased the myotube diameter (Figure 2C) and elevated the expressions of *Atrogin-1* and *MuRF-1* in Dex-treated C2C12 myotubes (Figure 2D), suggesting their functional role in resisting atrophy in C2C12 myotubes. Thus, *GR* is required for Dex to increase the miR-322 expression in C2C12 myotubes and contributes to Dex-induced atrophy in C2C12 myotube.

### 2.3. miR-322 Aggravated Dex-Induced Atrophy in C2C12 Myotubes

**Figure 3:**
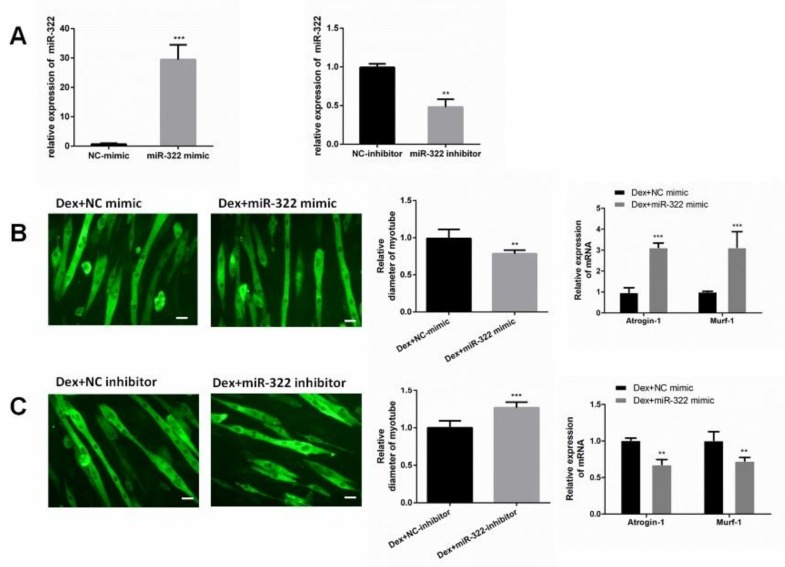
miR-322 aggravated Dex-induced atrophy in C2C12 myotubes. (**A**) miR-322 mimic increased the miR-322 expression, and miR-322 inhibitor decreased the miR-322 expression in C2C12 myotubes. (**B**) Immunofluorescent staining for *MyHC* in C2C12 myotubes showed the reduced myotube diameter and increased *Atrogin-1* and *MuRF-1* expression levels after transfection with miR-322 mimic in C2C12 myotubes. (**C**) Immunofluorescent staining for *MyHC* in C2C12 myotubes showed the increased myotube diameter and inhibited *Atrogin-1* and *MuRF-1* expressions after transfection with miR-322 inhibitor in C2C12 myotubes. Scale bar, 10 µm. Con, control. Dex, dexamethasone. ** *p* < 0.01 and *** *p* < 0.001.

To determine the role of miR-322 in Dex-treated C2C12 myotubes, miR-322 mimic or miR-322 inhibitor was used to increase or decrease the miR-322 expression in C2C12 myotubes, respectively (Figure 3A). Our results showed that miR-322 overexpression reduced the Dex-induced myotube diameter, accompanied with increased expressions of *Atrogin-1* and *MuRF-1* (Figure 3B). However, miR-322 inhibitor showed the opposite effects in Dex-treated C2C12 myotubes (Figure 3C). Thus, miR-322 aggravated Dex-induced atrophy in C2C12 myotubes.

### 2.4. miR-322 Induced Muscle Atrophy In Vitro Without Dex-Treated

**Figure 4:**
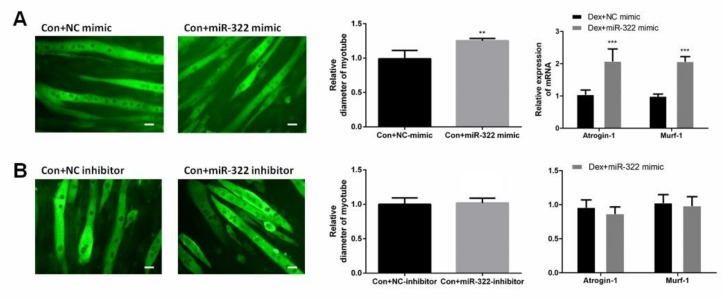
miR-322 induced muscle atrophy in vitro. (**A**) Immunofluorescent staining for *MyHC* in C2C12 myotubes showed reduced myotube diameter, accompanied with increased *Atrogin-1* and *MuRF-1* expression after transfection with miR-322 mimic. (**B**) Immunofluorescent staining for *MyHC* in C2C12 myotubes displayed no significant change in the myotube diameter and expressions of *Atrogin-1* and *MuRF-1* after transfection with miR-322 inhibitor. Scale bar, 10 µm. Con, control. Dex, dexamethasone. ** *p* < 0.01 and *** *p* < 0.001.

miR-322 mimic or miR-322 inhibitor was transfected into C2C12 myotubes to study the effect of miR-322 on atrophy in C2C12 myotubes without Dex treatment. miR-322 overexpression reduced the myotube diameter, accompanied with the increased expressions of miR-322 *Atrogin-1* and *MuRF-1* in C2C12 myotubes (Figure 4A). This result suggested that miR-322 induced atrophy in C2C12 myotubes without Dex treatment. However, miR-322 inhibitor showed no significant effects on C2C12 myotubes (Figure 4B).

### 2.5. IGF1R and INSR Are Target Genes of miR-322

**Figure 5:**
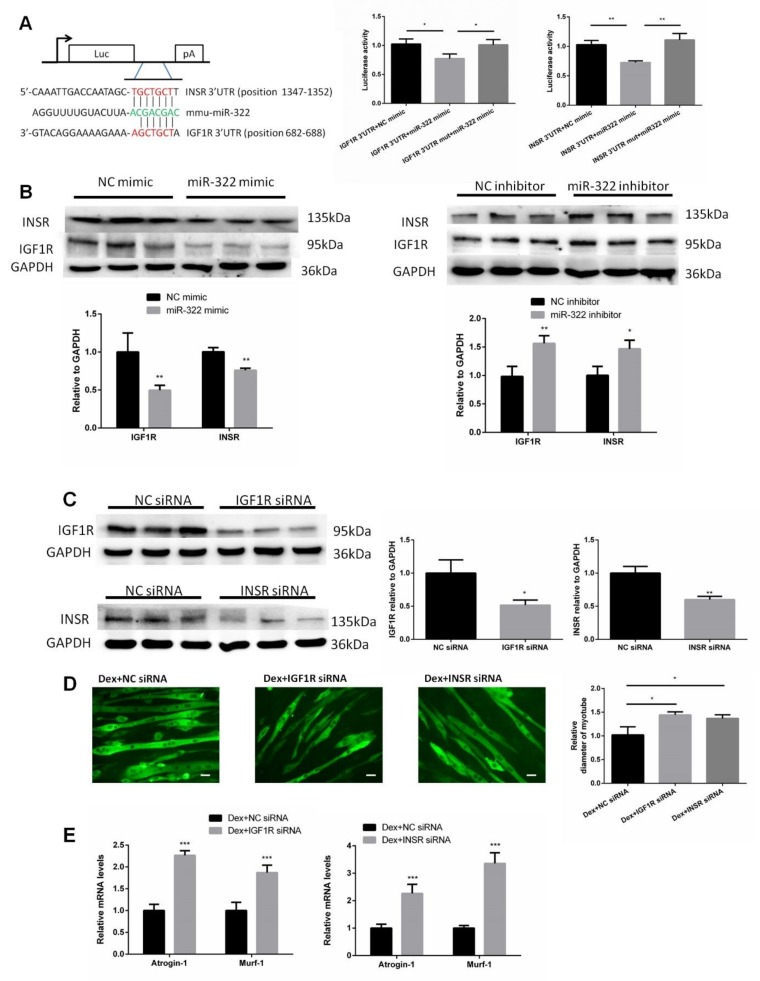
Insulin-like growth factor 1 receptor (*IGF1R*) and insulin receptor (*INSR*) of mice were identified as two target genes of miR-322. (**A**) TargetScan and luciferase reporter assay showed that *IGF1R* and *INSR* are two direct target genes of miR-322. (**B**) Western blot showed that miR-322 negatively regulated the expressions of *IGF1R* and *INSR* in the C2C12 myotubes. (**C**) The expressions of *IGF1R* and *INSR* were inhibited by siRNAs. (**D**) Immunofluorescent staining for *MyHC* in C2C12 myotubes showed that the inhibited expressions of *IGF1R* and *INSR* induced atrophy in the C2C12 myotubes. (**E**) qPCR analysis showed the inhibited expressions of *IGF1R* and *INSR* induced by the upregulation of *Atrogin-1* and *MuRF-1* in C2C12 myotubes. Scale bar, 10 µm. NC, negative control. Dex, dexamethasone. * *p* < 0.05, ** *p* < 0.01, and *** *p* < 0.001.

*IGF1R* and *INSR* of mice were predicted as putative target genes of miR-322 and used to investigate the mechanism by which miR-322 promotes muscle atrophy by using the bioinformatic tool TargetScan. *IGFR* and *INSR* were selected as the target genes of miR-322 due to their important roles in the growth and development of skeletal muscle. We then cloned the 3′UTRs of *IGF1R* and *INSR*, which included the binding sites of miR-322, into separate plasmids. Luciferase assays showed that exogenous miR-322 reduced the luciferase activity in cells transfected with the construct with 3′UTR of either *IGF1R* or *INSR* but showed no effect when the putative miR-322 binding sites of either *IGF1R* or *INSR* 3′UTR was mutated (Figure 5A). Transfection of miR-322 mimic into C2C12 myotubes resulted in decreased protein levels of *IGF1R* and *INSR*. Conversely, transfection with miR-322 inhibitor resulted in increased expressions of *IGF1R* and *INSR* (Figure 5B). To further assess whether *IGF1R* and *INSR* mediate atrophy in Dex-induced C2C12 myotubes, *IGF1R* siRNA, or *INSR* siRNA was used to inhibit the expression of *IGF1R* or *INSR*, respectively (Figure 5C). We observed that either knock-down of *IGF1R* or *INSR* induced atrophy in C2C12 myotubes, as determined by the myotube diameter and expression levels of *Atrogin-1* and *MuRF-1* (Figure 5E). These results indicated that *IGF1R* and *INSR* played key roles in miR-322 contributing to the atrophy in Dex-induced C2C12 myotubes.

### 2.6. miR-322 Contributed to Dex-Induced Muscle Atrophy In Vivo

**Figure 6:**
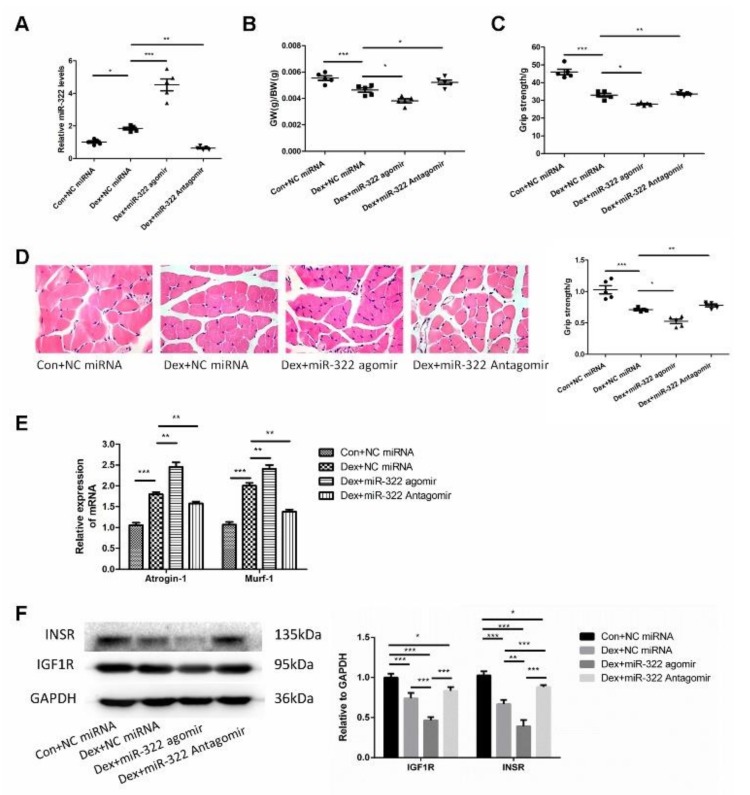
miR-322 aggravated Dex-induced muscle atrophy in vivo. (**A**) qRT-PCR analysis showed the expression of miR-322 (**B**) Gastrocnemius weight/body weight (GW/BW) ratio in mice was showed. (**C**) Grip strength of the right hind limb was showed. (**D**) HE staining (400×) showed muscle fiber diameter in mice. (**E**) qRT-PCR analysis showed *Atrogin-1* and *MuRF-1* expression levels in mice. (**F**) Western blot analysis showed increased expression of *IGF1R* and *INSR* in vivo. Scale bar, 10 µm. NC, negative control. Dex, dexamethasone. * *p* < 0.05, ** *p* < 0.01 and *** *p* < 0.001.

Dex was used to induce muscle atrophy in the gastrocnemius muscles of mice to characterize the in vivo characteristics of miR-322 in Dex-induced muscle atrophy. miR-322 agomir or miR-322 antagomir were used to increase or inhibit the miR-322 expression in mouse gastrocnemius muscles treated with Dex, respectively(Figure 6A). Interestingly, miR-322 expression was increased in the gastrocnemius muscles of mice treated with Dex (Figure 6A), which was consistent with the results in C2C12 myotube. miR-322 agomir or miR-322 antagomir on gastrocnemius muscle atrophy was supported by the following experimental data. Gastrocnemius weight/body weight (GW/BW) and grip strength were decreased by miR-322 agomir and increased by miR-322 antagomir (Figure 6B,C). Hematoxylin-eosin staining (HE staining) showed that muscle fiber diameter decreased. However, miR-322 antagomir showed the opposite effect to miR-322 agomir in the gastrocnemius muscles of mice (Figure 6D). Meanwhile, the expressions of atrogenes *Atrogin-1* and *MuRF-1* were increased by miR-322 agomir and inhibited by miR-322 antagomir (Figure 6E). These data indicate that miR-322 contributed to Dex-induced muscle atrophy, whereas miR-322 inhibition attenuated Dex-induced muscle atrophy in vivo. In addition, the expression levels of *IGF1R* and *INSR*, which were identified as the target genes of miR-322 in C2C12 myotubes, were inhibited by miR-322 agomir and by Dex in vivo. The expression level of *INSR* was increased by miR-322 antagomir (Figure 6F). This suggested that *IGF1R* and *INSR* were the target genes of miR-322 in vivo.

## 3. Discussion

Dex has been considered an effective agent to induce muscle atrophy in vitro or in vivo given its potential to stimulate protein catabolism [26]. This property might result from multiple mechanisms, including the inhibition of amino acid transport (leucine in particular), disrupted protein synthesis by inhibition of the activities of insulin and *IGF1*, and inhibition of myogenesis through myogenin downregulation [8]. Recent studies demonstrated that microRNAs play key roles in skeletal muscle atrophy [27,28]. miRNAs participate in multiple regulatory pathways in the skeletal muscle [29]. Accumulating evidence suggests that the aberrant expression levels of miRNAs, such as miR-1, miR-133, miR-23a, miR-206, miR-27, miR-628, miR-431, and miR-21 [20,21,30,31,32,33,34], contribute to muscle atrophy in a variety of animal models. In this study, we noted that miR-322 elevated and aggravated Dex-induced muscle atrophy by targeting IGF1R and *INSR*. Our results identify miR-322 as a novel target for promising therapeutic candidates. Meanwhile, Dex also causes other passive effects, including hypertension, osteoporosis, and depression [4]. Whether miR-322 reduces these side effects needs to be further explored. In addition, muscle atrophy can be commonly induced by a variety of stress conditions, such as acute and chronic inflammation, cancer cachexia, and starvation, that stimulate protein degradation [35,36]. Further research is still needed to determine if miR-322 exerts any effect on muscle atrophy induced by other causes.

miR-322 regulates multiple fundamental cellular processes, including cell cycle and cell differentiation, and paradoxically participates in tumor initiation and progression [23]. Based on bioinformatics analysis and further experimental validation, we identified *IGF1R* and *INSR* as target genes of miR-322. *IGF1R* and *INSR* are recognized as critical factors for controlling the balance between protein synthesis and degradation [37,38]. In addition, deactivation of *IGF1R* and *INSR* would result in decreased protein synthesis and increased protein degradation, possibly leading to muscle atrophy [39,40] Our data were consistent with those of a previous study indicating miR-322 as a regulator of *IGF1R* and *INSR* in vitro and in vivo in the heart of mice [24] We also observed the decreased expressions of *IGF1R* and *INSR* in Dex-treated mice. Furthermore, the inhibition of *IGF1R* or *INSR* by siRNA aggravated Dex-induced muscle atrophy, and this effect was consistent with miR-322 overexpression. Collectively, *IGF1R* and *INSR* serve as key target genes of miR-322 in Dex-induced C2C12 myotubes. Other target genes of miR-322 also play important roles in muscle development and regeneration. Previous studies have shown that miR-322 represses skeletal muscle differentiation by regulating rRNA synthesis by inhibiting Pol I pre-initiation complex formation [23,41]. Other target genes of miR-322 should be identified to help us understand the role of miR-322 in muscle atrophy.

The *GR* is mandatory for muscle atrophy in response to excess glucocorticoid (*GC*) both in vitro [42] and in vivo [43]. In addition, the muscle-specific inhibition of *GR* was resistant to the atrophy induced by Dex in mice [43]. In this study, *GR* inhibition eradicated the effects of Dex on muscle atrophy in C2C12 myotubes. These results support *GR* as an important mediator of skeletal muscle atrophy and associated gene expression in response to Dex. We also noted that *GR* knockdown with siRNA inhibited the increase in miR-322 and muscle atrophy in Dex-induced myotubes. This finding suggests that *GR*, as a ligand-activated transcription factor lays key roles in aggravating Dex-induced atrophy by miR-322 [44]. Interestingly, miR-322 inhibition could attenuate Dex-induced atrophy in C2C12 myotubes. Meanwhile, miR-322 inhibition showed no evident effects on C2C12 myotubes without Dex treatment or the expression of *IGF1R* in vivo. This result could be explained as follows. First, the effect of miR-322 inhibitor on the miR-322 expression was less than that of miR-322 mimics. Second, miR-322 worked by affecting the expression of its target genes, which are regulated by various factors. In other words, miR-322 is not a determinant of cellular molecular processes, and its effects are easily influenced by the expression of other genes. For example, miR-322 and its target protein Tob2 form a regulatory circuit to modulate Osterix mRNA stability in osteoblast. These studies showed that miR-322 interacts with other genes to exert its functions. To make miR-322 a therapeutic target for Dex-induced muscle atrophy, we need to gain further insights into how miR-322 works with other genes.

## 4. Materials and Methods

### 4.1. Cell Culture and Transfection

C2C12 cells (mouse skeletal myoblasts) were cultured in growth media (Dulbecco’s modified Eagle’s medium [DMEM]) containing 4.5 g L^−1^ glucose plus 10% fetal bovine serum (Atlanta Biologicals, Lawrenceville, GA, USA) and 1% penicillin and streptomycin at 37 °C with 5% CO_2_. C2C12 myoblasts were planted in 12-well plate until 80% confluence to induce differentiation. Then, C2C12 myoblasts were differentiated into myotubes by replacing growth media with differentiation media (DMEM supplemented with 2% fetal bovine serum and 1% penicillin and streptomycin). After four days, multinuclear myotubes were formed.

The formed C2C12 myotubes were treated with atrophy medium (50 µM Dex) or differentiation media added with PBS; the volume of Dex served as the control. After incubation for 24 h, the cells were harvested or used for morphological analysis.

The mimic negative control (NC), miR-322 mimic, inhibitor NC, miR-322 inhibitor, *GR* siRNA, *INSR* siRNA and *IGF1R* siRNA were synthesized by GenePharma (Suzhou, China) and transfected into C2C12 myotubes with a LipoPlus™ reagent (Sage creation, Beijing, China), respectively, according to the manufacturer’s instructions. After 12 h, the medium of the transfected myotubes was switched to atrophy medium for 24 h as described above.

### 4.2. Luciferase Reporter Assays

Fragments of IGF1 receptor (*IGF1R*) 3′-UTR and insulin receptor (*INSR*) 3′-UTR containing putative miR-322 binding sites or mutational miR-322 binding sites were synthesized by Genewiz (Suzhou, China) and cloned into the psiCHECK2 vector (Promega, Madison, WI, USA) between the XhoI and NotI sites, respectively. These fragments are listed in Table 1. All constructs were verified by DNA sequencing by Genewiz (Suzhou, China). HEK293T was transfected with 200 ng psiCHECK2-IGF1R-WT or psiCHECK2-IGF1R-Mut and 50 nM miR-322 mimic or mimic NC by using Lipofectamine 3000 Reagent in 96-well plates for 48 h. The activation of firefly and Renilla luciferase was analyzed by a dual-luciferase reporter assay kit (Promega) according to the manufacturer’s instructions.

### 4.3. Animal Experiments

Eight-week-old male C57BL/6 mice were purchased from the Liaoning Changsheng Biotechnology Co., Ltd. (ShenYang, China). The mice were provided with standard pellet diet and water ad libitum and were maintained under a 12 h light/12 h dark cycle at room temperature. All animal welfare and experimental procedures were performed strictly according to the guidelines from the National Institutes of Health Guide for the Care and Use of Laboratory Animals (NIH Publications No. 8023, revised 1978). Additionally, the procedures were reviewed and approved by the Institutional Animal Care and Use Committee of Jilin University, approval code: SY201907001; approval Date: 18 August 2019).

The mice were divided into four groups, A, B, C, and D. The agomir and antagomir of miR-322 were synthesized by GenePharma (Suzhou, China) and used to increase or decrease miR-322 expression in vivo, respectively. Mice in group A were treated with phosphate-buffered saline (PBS) and negative control (NC) miRNA (25 nmol per mice). Mice in group B were treated with Dex (25 mg kg^−1^ per day) and NC miRNA (25 nmol per mice). Mice in group C were treated with Dex (25 mg kg^−1^ per day) and miR-322 agomir (25 nmol per mice). Mice in group D were treated with Dex (25 mg kg^−1^ per day) and miR-322 antagomir (25 nmol per mice). All the mice were treated by intravenous injection once a day for seven days. All mice were killed after one week.

### 4.4. Quantitative Real-Time Polymerase Chain Reaction (qPCR)

Total RNA extraction from muscles and cells was performed by a Trizol reagent (Invitrogen, Carlsbad, CA, USA) according to the manufacturer’s instructions. Then, total RNA was reverse-transcribed using the PrimeScript™ RT reagent kit with gDNA Eraser (Perfect Real Time) (Takara Biotech, Co., Ltd., Dalian, China), and qPCR was performed with the TB Green^®^ Premix Ex Taq™ II (Tli RNaseH Plus) (Takara Biotech. Co., Ltd., Dalian, China). Glyceraldehyde 3-phosphate dehydrogenase was used as an internal control. The primer sequences used in this study were consistent with those of previous studies [24]. For qPCR of miRNA, RNA was reverse-transcribed using a stem-loop primer as described elsewhere, and U6 was used as an internal control [45]. The relative expression level was calculated using the 2^−ΔΔ*C*t^ method.

### 4.5. Western Blot

Protein samples were extracted from muscles or cells by using radioimmunoprecipitation assay buffer (KeyGEN, NanJing, China). The concentration of protein sample was determined by the BCA Protein Assay Kit (KeyGEN, NanJing, China). Equal amounts of protein samples were separated by 10% sodium dodecyl sulfate–polyacrylamide gel electrophoresis gels and transferred to polyvinylidene difluoride (PVDF) membranes. Then, the membranes were blocked with 10% nonfat-dried milk for 1 h at room temperature. Primary antibodies were incubated, followed by a horseradish peroxidase-conjugated secondary antibody. The primary antibodies used were as follows: myosin heavy chain (MyHC, 1:1000, R&D, Minnesota, USA), IGF1R (1:1000, Bioworld Technology, Inc., Minnesota, USA), INSR (1:1000, Cell Signaling Technology, Inc, Beverly, MA, USA), and GR (1:1000, Proteintech, Inc., Chicago, USA). Subsequently, the polyvinylidene difluoride (PVDF) membranes were visualized using commercial electrochemiluminescence (ECL) kits (Cell Signaling Technology, Beverly, MA, USA).

### 4.6. Staining

Gastrocnemius muscle samples were freshly isolated and immersed in 4% paraformaldehyde (PFA) (Solarbio, BeiJing, China). Transverse sections of muscle tissues with 10 mm thickness were subjected to HE staining by using a commercial kit (KeyGEN, NanJing, China) according to the manufacturer’s instruction.

To determine the diameter of myotubes in vitro, C2C12 myotubes were fixed by 4% PFA for 30 min at room temperature, permeabilized with 0.5% Triton X-100 for 20 min, blocked with 5% bovine serum albumin in PBS with Tween 20 PBST for 1 h at room temperature, and incubated with Primary antibodie of myosin heavy chain (MyHC, 1:100, R&D, USA) diluted in prostate specific antigen overnight at 4 °C. Then, the myotubes were incubated with secondary antibody goat anti-mouse IgG (H+L)-FITC (1:200, Bioworld, NanJing, China) for 1 h at room temperature. Nuclear staining was performed with 4′,6-diamidino-2-phenylindole. Images were captured by a fluorescence microscope (Leica, Frankfurt, Germany), and the diameter of myotubes was measured by Image J 2x (Rawak Software Inc., Stuttgart, Germany).

### 4.7. Grip-Strength Test

A digital grip-strength meter (YLS-13A, Yiyan Technology Co., Ltd., HongKong, China) was used to measure the grip strength of mice according to a known protocol [46]. In brief, the mice were acclimated to the environment for at least 10 min before the start of the test. The mice were allowed to grab the metal pull bar and were pulled backward until they let go. The force at the time of release was recorded. Each mouse was tested five times with a 30 s break between tests.

### 4.8. Statistical Analysis

The results were presented as the mean ± standard deviation from at least three independent experiments. The difference between groups was analyzed using Student t-test when comparing only two groups or one-way analysis of variance when comparing more than two groups. All analyses were performed using GraphPad Prism 6.0. Differences were considered significant with *p* < 0.05 (* *p* < 0.05, ** *p* < 0.01, and *** *p* < 0.001).

## Figures and Tables

**Table 1:** The 3′-untranslated region (3′-UTR) sequences that was cloned into psiCHECK2.

psiCHECK2-IGF1R-WT	CTCGAGATCTATACATCTGTACAGGAAAAGAAAA-*GCTGCT*-ATTTTTTTTTTGTTCTTTATCTTTGTGGATTTAATCTATGAA GCGGCCGC
psiCHECK2-IGF1R-Mut	CTCGAGATCTATACATCTGTACAGGAAAAGAAAA-*GGTCCA*-ATTTTTTTTTTGTTCTTTATCTTTGTGGATTTAATCTATGAA GCGGCCGC
psiCHECK2-INSR-WT	CTCGAGACAAAATCAGTTCCTCAAATTGACCAATAGC-*TGCTGC*-TTTCATATTTTATTTTGGGAAAGGGTGTGTATTCCTAAG GCGGCCGC
psiCHECK2-INSR-Mut	CTCGAGACAAAATCAGTTCCTCAAATTGACCAATAGC-*TCCAGG*-TTTCATATTTTATTTTGGGAAAGGGTGTGTATTCCTAAG GCGGCCGC

The italics represent the binding sites of miR-322.

## Supplementary Materials

Supplementary File 1

## Abbreviations

Dex	Dexamethasone
IGF1R	Insulin-like growth factor 1 receptor
INSR	Insulin receptor
GR	Glucocorticoid receptor
HE	Hematoxylin eosin
WT	Wild-type
MyHC	Myosin heavy chain
